# Statement for gastroesophageal reflux disease after peroral endoscopic myotomy from an international multicenter experience

**DOI:** 10.1007/s10388-019-00689-6

**Published:** 2019-09-26

**Authors:** Haruhiro Inoue, Hironari Shiwaku, Yasutoshi Kobayashi, Philip W. Y. Chiu, Robert H. Hawes, Horst Neuhaus, Guido Costamagna, Stavros N. Stavropoulos, Norio Fukami, Stefan Seewald, Manabu Onimaru, Hitomi Minami, Shinwa Tanaka, Yuto Shimamura, Esperanza Grace Santi, Kevin Grimes, Hisao Tajiri

**Affiliations:** 1 Digestive Diseases Center, Showa University Koto-Toyosu Hospital, Toyosu 5-1-38, Koto-Ku, Tokyo, 135-8577 Japan; 2 Department of Gastroenterological Surgery, Fukuoka University Faculty of Medicine, 7-45-1, Nanakuma, Jyonan-ku, Fukuoka, 814-0180 Japan; 3 Department of Gastroenterology and Hepatology, Jichi Medical University, Shimotsuke, Tochigi Japan; 4 The Institute of Digestive Disease, Faculty of Medicine of the Chinese University of Hong Kong, Hong Kong, China; 5 Center for Interventional Endoscopy, Florida Hospital Orlando, Orlando, Florida USA; 6 Department of Internal Medicine, Evangelisches Krankenhaus Düsseldorf, Düsseldorf, Germany; 7 Digestive Endoscopy Unit, Fondazione Policlinico Universitario A. Gemelli IRCCS, Catholic University, Rome, Italy; 8 Division of Gastroenterology, Hepatology, and Nutrition, NYU-Winthrop Hospital, New York, USA; 9 Division of Gastroenterology and Hepatology, Mayo Clinic Arizona, Scottsdale, AZ USA; 10 Centre of Gastroenterology, Klinik Hirslanden, Zurich, Switzerland; 11 Department of Gastroenterology and Hepatology, Nagasaki University, Nagasaki, Japan; 12 Division of Gastroenterology, Department of Internal Medicine, Graduate School of Medicine, Kobe University, Kobe, Japan; 13 Section of Gastroenterology and Digestive Endoscopy, De La Salle University Medical Center, Dasmarinas City, Philippines; 14 Department of Surgery, University of Cincinnati College of Medicine, Cincinnati, OH USA; 15 Department of Innovative Interventional Endoscopy Research, The Jikei University School of Medicine, Tokyo, Japan

**Keywords:** Achalasia, GERD, Myotomy

## Abstract

It has been 10 years since peroral endoscopic myotomy (POEM) was reported for the first time, and POEM has currently become the standard treatment for achalasia and related disorders globally because it is less invasive and has a higher curative effect than conventional therapeutic methods. However, there are limited studies comparing the long-term outcomes of POEM with those of conventional therapeutic methods, particularly in the occurrence of gastroesophageal reflux disease (GERD) after therapy. With this background, we held a consensus meeting to discuss the pathophysiology and management of GERD after POEM based on published papers and experiences of each expert and to discuss the prevention of GERD and dealing with anti-acid drug refractory GERD. This meeting was held on April 27, 2018 in Tokyo to establish statements and finalize the recommendations using the modified Delphi method. This manuscript presents eight statements regarding GERD after POEM.

## Introduction

It has been 10 years since peroral endoscopic myotomy (POEM) was reported for the first time; currently, POEM has become the standard treatment for achalasia and related disorders worldwide as it is less invasive and has a higher curative effect than conventional therapeutic methods [1, 2].

However, there are limited studies comparing the long-term outcomes of POEM with those of conventional therapeutic methods, particularly with respect to the occurrence of gastroesophageal reflux disease (GERD) after therapy [3].

During the ordinary procedure of Heller myotomy (laparotomy or laparoscopic), adjacent structures surrounding the distal esophagus responsible for natural antireflux mechanisms, including the phrenoesophageal ligament, are inevitably circumferentially dissected to perform myotomy on the esophagus. This procedure results in an impairment of the natural antireflux mechanisms and causes postoperative GERD. Therefore, Heller myotomy requires fundoplication to prevent GERD. Several studies showed that POEM achieved similar curative effect compared with Heller myotomy for achalasia [3–6]. However, a fundoplication is not performed during POEM. Therefore, when POEM was first introduced, the development of GERD was a huge concern. However, to date, there have been few reports on the occurrence of GERD requiring surgical intervention with fundoplication [7–10].

With this background, we held a consensus meeting to discuss the pathophysiology and management of GERD after POEM based on published papers and personal experiences from each expert, and to discuss how to prevent GERD and how to deal with GERD refractory to acid suppressing medications when it is encountered. This consensus meeting resulted in eight statements regarding GERD after POEM. This meeting was held on Friday, April 27, 2018, prior to the 3rd Tokyo International Endoscopy Live Course (Tokyo Live 2018).

## GERD after POEM–consensus statements development process–consensus statements committee members

The consensus statements committee comprised sixteen international gastrointestinal endoscopists, including five members of the development committee and fifteen members of the evaluation process as shown in Table 1. The development committee selected the following eight topics as targets for core statements:

**Table 1:** Committee members

Committee members for the development of statement		
1	Haruhiro Inoue	Digestive Diseases Center, Showa University Koto-Toyosu Hospital, Tokyo, Japan
2	Hironari Shiwaku	Department of Gastroenterological Surgery, Fukuoka University, Fukuoka, Japan
3	Yasutoshi Kobayashi	Department of Gastroenterology and Hepatology, Jichi Medical University, Tochigi, Japan
4	Manabu Onimaru	Digestive Diseases Center, Showa University Koto-Toyosu Hospital, Tokyo, Japan
5	Hitomi Minami	Department of Gastroenterology and Hepatology, Nagasaki University, Nagasaki, Japan
Committee members of the evaluation process		
1	Haruhiro Inoue	Digestive Diseases Center, Showa University Koto-Toyosu Hospital, Tokyo, Japan
2	Robert H. Hawes	Center for Interventional Endoscopy, Florida Hospital Orlando, Florida, USA
3	Horst Neuhaus	Department of Internal Medicine, Evangelisches Krankenhaus Düsseldorf, Düsseldorf, Germany
4	Guido Costamagna	Digestive Endoscopy Unit, Fondazione Policlinico Universitario A.Gemelli IRCCS, Catholic University, Rome, Italy
5	Stavros N. Stavropoulos	Division of Gastroenterology, Hepatology, and Nutrition, NYU-Winthrop Hospital, New York, USA
6	Philip W.Y. Chiu	The Institute of Digestive Disease, Faculty of Medicine of the Chinese University of Hong Kong, Hong Kong, China
7	Norio Fukami	Division of Gastroenterology and Hepatology, Mayo Clinic Arizona,Scottsdale, Arizona, USA
8	Stefan Seewald	Centre of Gastroenterology, Klinik Hirslanden, Zürich, Switzerland
9	Hironari Shiwaku	Department of Gastroenterological Surgery, Fukuoka University, Fukuoka, Japan
10	Manabu Onimaru	Digestive Diseases Center, Showa University Koto-Toyosu Hospital, Tokyo, Japan
11	Hitomi Minami	Department of Gastroenterology and Hepatology, Nagasaki University, Nagasaki, Japan
12	Shinwa Tanaka	Division of Gastroenterology, Department of Internal Medicine, Graduate School of Medicine, Kobe University, Kobe, Japan
13	Esperanza Grace Santi	Section of Gastroenterology and Digestive Endoscopy, De La Salle University Medical Center, Dasmarinas City, Philippines
14	Kevin Grimes	Department of Surgery, University of Cincinnati College of Medicine, Cincinnati, OH, USA
15	Hisao Tajiri	Department of Innovative Interventional Endoscopy Research, The Jikei University School of Medicine, Tokyo, Japan

(i) The incidence of GERD after POEM
(ii) The incidence of late complications of GERD after POEM
(iii) The rate of GERD after POEM and after Heller with partial fundoplication
(iv) The role of anti-acid drugs after POEM
(v) The rate of additional fundoplication for refractory GERD after POEM
(vi) The reasons for the occurrence of GERD after POEM
(vii) The usefulness of the double-scope transillumination technique during POEM as it relates to GERD minimization
(viii) Management for anti-acid drug refractory GERD after POEM.

## Evaluation process

The consensus statement development committee conducted a systematic review on the clinical questions (CQs) further mentioned in the text. Committee members independently searched literature pertinent to the CQs using Medline, Cochrane Library, and Japan Medical Abstract Society Database, starting from 2010 when the first case of POEM for humans was reported up to April 2018 and performed meta-analysis if applicable. After reviewing the final results of the systematic review and meta-analysis for the CQs, development committee members finalized the proposed guideline statements for each CQ assessing the quality of evidence and assigning a strength of recommendation in accordance with the Grading of Recommendations Assessment, Development, and Evaluation (GRADE) tool (Table 2) [11, 12]. The evaluation committee conducted the consensus meeting for GERD after POEM at Tokyo Live 2018 Satellite Symposium. Modified Delphi method was used to reach the consensus by the 15 expert panels. Delphi voting involves a rating scale (1–3, disagree; 4–6, unsure; 7–9, agree) and the results are expressed as the median, highest, and lowest [13]. At least 80% agreement was required for consensus to be reached. Where consensus could not be achieved, statements were revised and another Delphi round was performed. When these processes were complete, the committee released the final version of the manuscript.

**Table 2:** Evidence level and strength of recommendation

Grades of recommendation
1: Strong recommendation
2: Weak recommendation
N/A: Unclear recommendation, or recommendation grade cannot be determined
Evidence level
A: Based on strong evidence
B: Based on moderate evidence
C: Based on weak evidence
D: Based on very weak evidence

Regarding statements with evidence level D, they are written not as CQs but as future research questions (FRQs). The summary of CQ, FRQ and each statement is shown in Table 3.

**Table 3:** Summary of statement

	CQ and FRQ	Statement	Strength of recommendation	Evidence Level
CQ1	What is the incidence of GERD after POEM?	POEM may induce GERD, but incidence depends on measurement	N/A	B
CQ2	Are there any reports of stenosis, bleeding, or Barrett’s esophageal cancer due to GERD after POEM?	The incidence of late complications of GERD after POEM seems to be low; however, further long-term investigation is needed	N/A	C
CQ3	Is post-POEM GERD higher than GERD after laparoscopic Heller-Dor?	Based on current data, GERD after POEM occurs more frequently than after Heller plus partial fundoplication	N/A	C
FRQ 4	What is the role of proton pump inhibitor (PPI) after POEM?	Most patients with post-POEM GERD respond to PPI therapy; however, the indications for PPI are not well defined	N/A	D
FRQ 5	What is the rate of cases where additional fundoplication was performed for refractory GERD after POEM?	The need for fundoplication to treat GERD after POEM is extremely low	N/A	D
FRQ 6	Why is the rate of GERD high in POEM which preserves the periesophageal suspensory ligaments involved in natural antireflux mechanisms?	Excessive gastric myotomy and incision of the collar sling fibers may increase the frequency of GERD after POEM	N/A	D
FRQ 7	Is the double-scope transillumination method helpful for controlling the length of myotomy?	Currently, the double-scope method is the most reliable way to confirm the length and direction of myotomy on the gastric side	1	D
FRQ 8	How should the patient with medication refractory GERD after POEM be managed?	For refractory severe post-POEM GERD, some antireflux procedure may be considered	2	D

## Target patients

These consensus statements apply to patients who are considering POEM or underwent POEM. These consensus statements are intended to be used by the clinicians who engage in POEM practice and other clinicians providing aid for the digestive issues of patients undergoing POEM. These statements provide general recommendations regarding current standards of care for POEM procedures and, therefore, each statement’s users should carefully recognize that its application in clinical practice may need to be individualized according to each clinician’s background and resources, and patients’ background, preference, age, comorbidity, and social and medical conditions.

The consensus statements committee comprised sixteen international gastrointestinal endoscopists, including five members of the development committee and fifteen members of the evaluation process.

Recommendations can be categorized as strong, weak, or unclear.

Recommendations involve a trade-off between benefits and harms. Those making a recommendation should consider four main factors:

1. The trade-offs, considering the estimated size of the effect for the main outcomes, the confidence limits around those estimates, and the relative value placed on each outcome.
2. The quality of evidence
3. Translation of the evidence into practice in a specific setting, considering important factors that could modify the size of the expected effects, such as proximity to a hospital or availability of necessary expertise.
4. Uncertainty about baseline risk for the population of interest. If there is uncertainty about translating the evidence into practice in a specific setting, or uncertainty about baseline risk, this may lower our confidence in a recommendation.

Strong evidence: further research is unlikely to change our confidence in the estimate of effect.

Moderate evidence: further research is likely to have an important impact on our confidence in the estimate of effect and may change the estimate

Weak evidence: further research is extremely likely to have an important impact on our confidence in the estimate of effect and may change the estimate

Very weak evidence: any estimate of effect is extremely uncertain.

CQ1: what is the incidence of GERD after POEM?

Statement: POEM may induce GERD, but incidence depends on measurement.

Strength of recommendation: N/A

Evidence level: B

Delphi scores: Median = 8, Lowest = 7, Highest = 9

Commentary: according to several meta-analyses, symptomatic GERD after POEM occurred in 8.5–19% of patients [3, 14, 15]. The endoscopic findings of erosive esophagitis after POEM were detected in 13–29.4% of patients, whereas abnormal 24 pH study results were observed in 39–47.5% of patients [3, 14, 15]. In general, the rate of GERD after POEM depends on the type of measurement and there is significant difference between symptoms, endoscopic evidence, and pH measurement. In the future, a unified definition of GERD after POEM should be established to standardize the assessment of the incidence of GERD after POEM.

CQ2: are there any reports of stenosis, bleeding, or Barrett’s esophageal cancer due to GERD after POEM?

Statement: the incidence of late complications of GERD after POEM seems to be low; however, further long-term investigation is needed.

Delphi scores: Median = 8.5, Lowest = 7, Highest = 9

Strength of recommendation: N/A

Evidence level: C

Commentary: currently, there is no published report on the development of GERD with refractory stenosis, bleeding, or Barrett’s esophagus and carcinoma after POEM. However, there are verbal reports on a few cases of stenosis and bleeding due to GERD after POEM from high volume centers. In addition, although there were institutions that experienced the occurrence of SSBE (short segment Barrett Esophagus) associated with erosive esophagitis after POEM, there was no report on the occurrence of LSBE (long segment Barrett Esophagus). To date, Barrett’s related esophageal cancer after POEM has not been reported in the literature or by any of the expert centers. The panel recommended long-term follow-up and report of the clinical outcomes for patients with GERD after POEM to monitor GERD-related complications.

CQ3: is post-POEM GERD higher than GERD after laparoscopic Heller-Dor?

Statement: based on the current data, GERD after POEM occurs more frequently than after Heller plus partial fundoplication.

Strength of recommendation: N/A

Evidence level: C

Delphi scores: Median = 8, Lowest = 6, Highest = 9

Commentary: according to the meta-analyses by Schlottmann et al. and Repici et al., gastroesophageal reflux after POEM measured by pH monitoring was significantly higher than that of Heller–Dor operation [3, 14]. Other reports demonstrated no significant difference in pH monitoring between POEM and Heller myotomy [16].

The results from the studies which focused on endoscopic evidence of erosive esophagitis and symptomatic GERD comparing POEM and Heller myotomy are variable. Hence, there are no definitive conclusions [17, 18].

FRQ 4: what is the role of proton pump inhibitor (PPI) after POEM?

Statement: most patients with post-POEM GERD respond to PPI therapy; however, the indications for PPI are not well defined.

Strength of recommendation: N/A

Evidence level: D

Delphi scores: Median = 8, Lowest = 7, Highest = 9

Commentary: PPI is effective for the treatment of GERD after POEM [15]. According to a meta-analysis reported by Repici, the rate of PPI use ranged from 2.6 to 27.8% after POEM [14]. Most patients with post-POEM GERD respond to PPI therapy; however, the indication for use of PPIs after POEM is not well defined. Numerous studies reporting on the occurrence of GERD after POEM demonstrated that there is a discrepancy among the clinical symptoms of GERD, objective assessment with 24 h pH study, and endoscopic evidence of esophagitis [3, 14, 15]. Contrary to our general understanding that the prevalence of GERD symptoms in the general population is commonly higher than the rate of positive pH studies or the rate of endoscopic evidence of esophagitis, in achalasia patients receiving POEM, the pH study positivity and endoscopic esophagitis rates are usually higher than symptomatic reflux. This results in uncertainties regarding the use of PPIs after POEM, whether it is for symptomatic relief or healing esophagitis.

FRQ 5: what is the rate of cases where additional fundoplication was performed for refractory GERD after POEM?

Statement: the need for fundoplication to treat GERD after POEM is extremely low.

Strength of recommendation: N/A

Evidence level: D

Delphi scores: Median = 8, Lowest = 7, Highest = 9

Commentary: currently, there are a few reports which describe additional fundoplication for GERD after POEM [7–9]. Swanström states that the need for an antireflux procedure after POEM is exceedingly rare in his and other Western experiences [10]. According to multicenter retrospective study in Japan (Over 2000 cases), the rate of cases in which additional surgical fundoplication was performed for GERD after POEM was 0.1% (2 cases) (Unpublished data. Reported in this consensus meeting) [19].

FRQ 6: why is the rate of GERD high in POEM which preserves the periesophageal suspensory ligaments involved in natural antireflux mechanisms?

Statement: excessive gastric myotomy and incision of the collar sling fibers may increase the frequency of GERD after POEM.

Strength of recommendation: N/A

Evidence level: D

Delphi scores: Median = 8, Lowest = 6, Highest = 9

Commentary: in patients with achalasia, esophageal peristalsis affecting acid clearance is impaired. Therefore, even when performing Heller myotomy or performing POEM, postoperative GERD after incision of LES should be considered. Simic et al. reported that postoperative GERD occurs in 23.1% patients after Heller myotomy with full mobilization of abdominal esophagus around the hiatus [20]. Alternatively, preservation of the phrenoesophageal ligament during Heller myotomy can reduce the development of GERD, regardless of whether fundoplication was performed (GERD after Heller with limited hiatal dissection + Dor: 8.5%; GERD after Heller with limited hiatal dissection alone without fundopication: 9.1%). According to this theory, POEM which naturally preserves the phrenoesophageal ligament should reduce the development of GERD. However, the clinical rate of GERD after POEM remains high. Factors other than preservation of the phrenoesophageal ligament may have contributed to the occurrence of GERD after POEM [3, 14, 15]. According to the results of a multicenter retrospective study in Japan (Over 2000 cases, nonpublished data), it was suggested that excessive muscle incision of longer than 4 cm in the gastric cardia on the posterior side may be responsible for erosive esophagitis over Grade C (Unpublished data. Reported in this consensus meeting) [19]. Moreover, according to a single-center report by Fukuoka University, incision of the sling fibers (oblique muscle) at the gastric cardia may be the cause of significant GERD, leading to erosive esophagitis above Grade C (Unpublished data. Reported in this consensus meeting) [19]. From these results, the direction of myotomy is recommended anterior (1–2 o’clock) or posterior (4–5 o’clock) side which could preserve oblique muscle and the length of myotomy in gastric side is recommended 2–3 cm. Future studies should address the extent of myotomy, particularly to the gastric cardia, as well as the effect of anatomical dissection of the sling fibers on the occurrence of GERD after POEM.

FRQ 7: is the double-scope transillumination method helpful for controlling the length of myotomy?

Statement: currently, the double-scope method is the most reliable way to confirm the length and direction of myotomy on the gastric side.

Strength of recommendation: 1

Evidence level: D

Delphi scores: Median = 8, Lowest = 6, Highest = 9

**Fig. 1:**
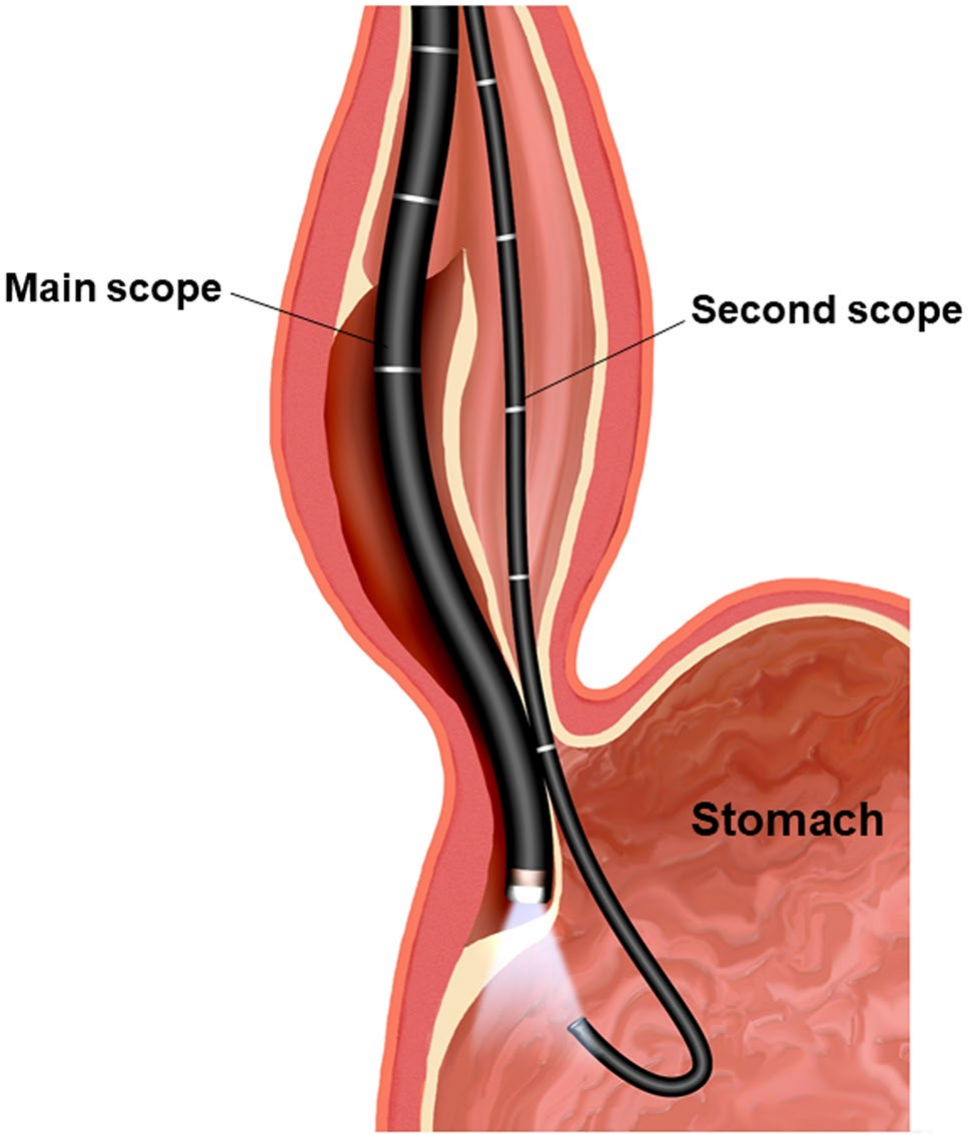
The image of double-scope method. A second endoscope was inserted into the stomach to examine the cardia region. If the procedure reached the gastric side, the light from the main scope within the submucosal space was visible through the second scope in the stomach

**Fig. 2:**
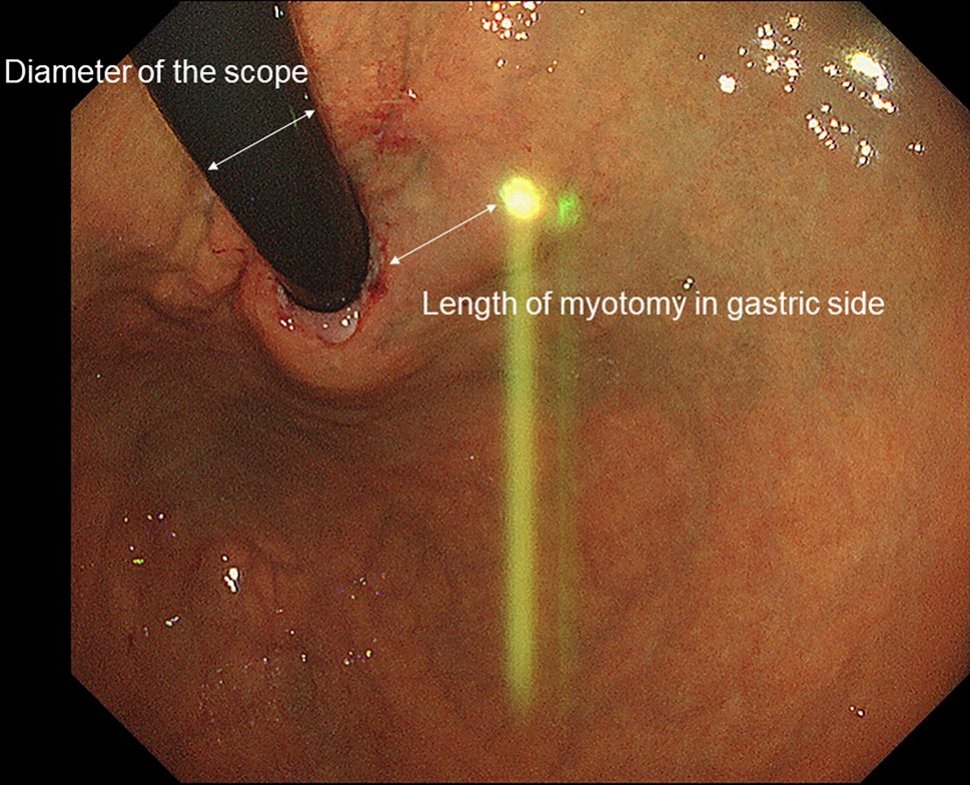
Endoscopic finding of double scope. The length of myotomy in gastric side can be measured based on the diameter of second scope

Commentary: originally, the double-scope method was proposed as a means to prevent incomplete myotomy of lower esophageal sphincter (LES) during POEM (Figs. 1 and 2) [21, 22]. The double-scope technique comprised observing the extent of submucosal tunnel development at the gastric cardia using an ultrathin endoscope while the original endoscope transilluminates through the mucosa while being at the end of the tunnel. Clinical outcomes from a multicenter retrospective study in Japan showed that excessive muscle incision of longer than 4 cm in the gastric cardia on the posterior side may result in the development of erosive esophagitis above LA Grade C after POEM (Unpublished data. Reported in this consensus meeting) [19]. Furthermore, according to the experience reported by Fukuoka University, preserving the oblique muscle may reduce the occurrence of GERD after POEM (Unpublished data. Reported in this consensus meeting) [19]. The double-scope technique is the most reliable method to confirm the length and direction of myotomy when observing the gastric cardia side. Therefore, it is recommended that operators should perform the double-scope method during POEM to confirm the extent of myotomy in the gastric cardia and reduce GERD after POEM.

CQ8: how should the patient with medication refractory GERD after POEM be managed?

Statement: for refractory severe post-POEM GERD, some antireflux procedures may be considered.

Strength of recommendation: 2

Evidence level: D

Delphi scores: Median = 7, Lowest = 5, Highest = 9

Commentary: most studies report the use of anti-acid therapy as first-line treatment for post-POEM GERD. Most cases respond to PPI [15]. Antireflux surgery will be considered for patients who have significant GERD symptoms and when PPI cannot completely relieve these symptoms. Antireflux surgery achieves control of gastroesophageal reflux through correction of local anatomical defects, including the damaged antireflux mechanisms such as the defective lower esophageal sphincter, oblique muscle and phrenoesophageal ligament. Published reports on antireflux surgery for the management of GERD after POEM are limited. POEM + F (POEM plus endoscopic fundoplication) may be another choice of treatment [23]. Currently, laparoscopic partial fundoplication (Dor or Toupet) is the most common surgical treatment and this is a routine antireflux procedure after laparoscopic Heller myotomy. According to a multicenter retrospective study in Japan (Over 2000 cases), the number of patients with severe GERD that required surgical fundoplication after POEM was 0.1% (2 cases) (Unpublished data. Reported in this consensus meeting) [19]. Although surgical fundoplication is an effective treatment for GERD, it is less than ideal to perform laparoscopic partial fundoplication after a successful endoscopic myotomy. In the future, research should focus on refining the techniques of POEM to minimize occurrence of GERD, as well as developing effective endoscopic antireflux procedures for the management of GERD after POEM.

## Conclusion

According to the results of this consensus meeting and the present published data, it was confirmed that most patients with post-POEM GERD respond to PPI therapy and fundoplication for refractory GERD is rarely needed in the decade-long POEM global experience. PPI remains the mainstay therapy for GERD after POEM with an extremely high rate of efficacy. There are limited data on the use of endoscopic antireflux procedures for the treatment of post-POEM GERD. Intriguing preliminary retrospective data from Japan suggest that POEM technique modifications that focus on limiting the length of myotomy and preserving the collar sling fibers may decrease the frequency of GERD after POEM. It is recommended that a multicenter prospective study evaluating these technique refinements should be considered in the near future.
